# Device-measured physical activity in adults born preterm with very low birth weight and mediation by motor abilities

**DOI:** 10.1371/journal.pone.0312875

**Published:** 2025-01-07

**Authors:** Silje Dahl Benum, Kristina Anna Djupvik Aakvik, Cathrin Vano Mehl, Atle Kongsvold, Stian Lydersen, Maria Vollsæter, Paul Jarle Mork, Eero Kajantie, Kari Anne I. Evensen

**Affiliations:** 1 Department of Clinical and Molecular Medicine, Norwegian University of Science and Technology, Trondheim, Norway; 2 Department of Public Health and Nursing, Norwegian University of Science and Technology, Trondheim, Norway; 3 Department of Mental Health, Regional Centre for Child and Youth Mental Health and Child Welfare, Norwegian University of Science and Technology, Trondheim, Norway; 4 Department of Pediatrics, Haukeland University Hospital, Bergen, Norway; 5 Department of Clinical Science, University of Bergen, Bergen, Norway; 6 Finnish Institute for Health and Welfare, Public Health Promotion Unit, Helsinki and Oulu, Finland; 7 Clinical Medicine Research Unit, MRC Oulu, Oulu University Hospital and University of Oulu, Oulu, Finland; 8 Children’s Hospital, Helsinki University Hospital and University of Helsinki, Helsinki, Finland; 9 Children’s Clinic, St. Olavs Hospital, Trondheim University Hospital, Trondheim, Norway; 10 Department of Rehabilitation Science and Health Technology, Oslo Metropolitan University, Oslo, Norway; Ulm University Medical Center, Department of Pediatrics and Adolescent Medicine: Universitatsklinikum Ulm Klinik fur Kinder- und Jugendmedizin, GERMANY

## Abstract

Physical activity (PA) is beneficial for several health outcomes. Adults born with very low birth weight (VLBW<1500g) undertake less PA than those born at term, have poorer motor abilities and may serve as a model on early life origins of PA. We therefore examined whether motor abilities mediate the association between being born with VLBW and device-measured PA. In a joint assessment of two longitudinal birth cohorts from Finland and Norway, PA was measured by two tri-axial accelerometers in 87 adults born preterm with VLBW and 109 controls born at term. We explored the mediating role of motor abilities assessed by standardized tests on the association between VLBW and device-measured PA. To do this, we examined group differences in metabolic equivalent of task (MET) min/day of moderate to vigorous PA (MVPA), light PA and sedentary. Analyses were adjusted for cohort, age and sex. MVPA was 40.4 (95% confidence interval [CI]: 13.3 to 69.4) MET min/day lower in the VLBW group than the control group. This was in part mediated through gross motor abilities, indicated by the indirect effect on the association between VLBW and MVPA being -15.6 (95% CI: -28.5 to -5.4) MET min/day. In conclusion, adults born preterm with VLBW undertake less MVPA than controls born at term, and gross motor abilities mediate this association. Interventions targeting motor abilities should be examined as potential ways to increase PA.

## Introduction

Physical activity (PA) is beneficial for several physical and mental health aspects [1]. International recommendations emphasize the importance of undertaking more moderate to vigorous PA (MVPA), and to reduce time spent in sedentary behaviors [1]. PA may be assessed by devices such as accelerometers, which are easy to administer and have been shown to have a high accuracy [2].

Being born preterm with very low birth weight (VLBW<1500g) is associated with adverse health outcomes which may appear at a young age and persist into adulthood [3, 4]. These include impaired lung function [5, 6], higher blood pressure [7], alterations in muscle mass and function [8] and body composition [9], reduced cardiorespiratory fitness [4], and lower exercise capacity [10] compared with controls born at term. The outcomes are accompanied by up to 50% lower levels of PA [10–14] by self-report, although these have not been confirmed by accelerometry [11, 12].

Motor difficulties have consistently been reported in several motor domains among individuals born preterm from childhood [15–17] to adulthood [18, 19]. This could possibly interfere with the ability to be physically active. Using data from two birth cohorts in Finland and Norway, we have shown that adults born with VLBW had poorer overall-, fine-, and gross motor abilities as compared with controls born at term [19]. In the general population, it is poorly understood why some people undertake more PA than others. Adults born preterm with VLBW could serve as a useful model in assessing motor abilities as one possible factor underlying PA. However, whether motor abilities mediate the association between VLBW and PA has not been investigated.

The aim of this study was therefore to examine whether motor abilities mediate the association between being born with VLBW and device-measured PA.

## Methods

### Study design

Data were collected with harmonized methods in two longitudinal birth cohorts; the Helsinki Study of Very Low Birth Weight Adults (HeSVA), Finland, and the Norwegian University of Science and Technology Low Birth Weight in a Lifetime Perspective (NTNU LBW Life), Norway. In the HeSVA study, infants born with VLBW (<1500g) discharged from the neonatal intensive care unit (NICU) at the Children’s Hospital in Helsinki between 1978 and 1985 were included [7]. Age- and sex-matched controls born at term (gestational age ≥37 weeks) not small for gestational age (birth weight for gestational age ≥-2SD) were recruited during a study visit in 2004–05. In the NTNU LBW Life study, VLBW infants with birth weight ≤1500g admitted to the NICU at the University Hospital in Trondheim between 1986 and 1988 were included [3]. Controls born at term not small for gestational age (birth weight for gestational age ≥10^th^ percentile, corrected for sex and parity) were recruited from a multicenter study enrolling pregnant women in 1986–88. Accelerometer data was collected from September 2019 to January 2021 as part of a larger data collection including body composition [9], motor abilities [19] and visual function [20].

### Study groups

**Fig 1:**
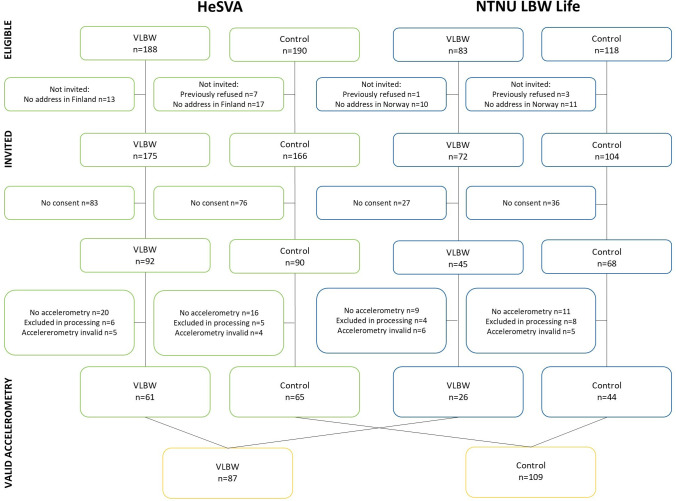
Flow chart of participants. Abbreviations: HeSVA = Helsinki Study of Very Low Birth Weight Adults; NTNU LBW Life = Norwegian University of Science and Technology Low Birth Weight in a Lifetime Perspective; VLBW = very low birth weight.

Flow of eligible and invited participants from HeSVA and NTNU LBW Life are shown in Fig 1. Of the 92 invited individuals born with VLBW from HeSVA who consented to participate, 20 did not wear accelerometers, 6 were excluded in data processing and 5 had invalid accelerometry data, resulting in 61 participants with valid accelerometer data in the VLBW group. Of the 45 invited individuals born with VLBW from NTNU LBW Life who consented to participate, 9 did not wear accelerometers, 4 were excluded in data processing and 6 had invalid accelerometry data, leaving 26 participants with valid accelerometer data in the VLBW group.

Of the 90 invited control participants from HeSVA who consented to participate, 16 did not wear accelerometers, 5 were excluded in data processing and 4 had invalid accelerometry data, resulting in 65 controls with valid accelerometer data. Of the 68 invited controls from NTNU LBW Life who consented to participate, 11 did not wear accelerometers, 8 were excluded in data processing and 5 had invalid accelerometry data, leaving 44 controls with valid accelerometer data. This resulted in 87 participants born with VLBW and 109 controls with valid accelerometry data.

### Background and clinical characteristics

Background characteristics were retrieved from hospital records and included maternal age at delivery, gestational age, birth weight, birth head circumference, Apgar score after 1 and 5 min, days of stay in NICU, days on ventilator, sepsis at birth, presence of interventricular hemorrhage on neonatal cerebral ultrasound, respiratory distress syndrome and bronchopulmonary dysplasia (BPD) defined as oxygen supply for ≥28 days. Information of maternal education was categorized into four education levels (*Basic or less*, *Secondary*, *Lower-level tertiary* or *Upper-level tertiary*).

Weight was measured in kg by a Seca medical Body Composition Analyzer (Seca® mBCA515, Hamburg, Germany) or a Seca electronic weight, and height was measured in cm. Body mass index (kg/m^2^) was calculated. Presence of cerebral palsy (CP) was assessed by self-report at the current follow up in HeSVA and diagnosed by a clinician at 14 years or by self-report at the current follow up in NTNU LBW Life. Neurosensory impairment (NSI) was defined as having CP, blindness, deafness (HeSVA) or hearing aid (NTNU LBW Life) and/or intellectual disability defined by self-report in young adulthood (HeSVA) or clinically assessed intellectual quotient <70 at 19, 14 or 5 years of age (NTNU LBW Life). Data on maternal and paternal education was collected in young adulthood (HeSVA) and at the 14- or 19-year follow-up (NTNU LBW Life) and were categorized into four education levels (*Basic or less*, *Secondary*, *Lower-level tertiary* or *Upper-level tertiary*) based on the International Standard Classification of Education (ISCED). Working hours/week was calculated based on working hours/day during the monitoring period measured by self-report.

### Outcome measures

#### Device-measured physical activity

Two tri-axial AX3 accelerometers (Axivity, Ltd. Newcastle, United Kingdom), administered by trained examiners, were used to measure PA [21]. One accelerometer was placed on the front of the right thigh approximately 10 cm above the upper border of the patella, and the other centrally on the lower back on the third lumbar segment. The accelerometers were attached to the skin using two adhesive films and double-sided tape and the participants were instructed to behave habitually during the monitoring period.

The accelerometers were configured to record at 50 Hz with a bandwidth of 8G using the OmGui software (version 1.0.0.37; Open Movement, Newcastle University, United Kingdom). Participants in NTNU LBW Life were instructed to remove the sensors at day eight of recording at the latest, and as the first and last day of monitoring were removed (i.e., the days of mounting and removing the accelerometers), they had a maximum of six recording days. Participants in HeSVA were instructed to remove the sensors at day nine of recording at the latest and had a maximum of seven recording days when the first and last day were removed.

Raw data was stored on the 512 MB internal flash drive of the accelerometers and later downloaded for further analysis. The file from each accelerometer was synchronized and combined into one CSV file and segmented into 5 second windows. The data was fed into an eXtreme Gradient Boosting (XGBoost) machine learning model trained to predict eight PA behaviors; running, cycling, brisk walking, moderate walking, slow walking, standing, sitting and lying [22, 23]. The machine learning model has been validated showing an overall accuracy of 96% in predicting the behaviors in free-living conditions [22, 23]. No-wear time was predicted by a separate XGBoost machine learning model. Only days with >23 hours of recordings were included in the analyses. Participants wearing only one accelerometer, who had error in synchronization of the sensor signals or who had more than one hour of no-wear time and thereby no recording days, were excluded in data processing by the machine learning model.

A manual check was done on the remaining data. An inclusion criterion was that the participants had to be able to walk independently. As the machine learning model did not detect all participants who wore only one sensor, these were excluded. To be considered a valid recording day >5 min in any dynamic activity or combination of activities (i.e. running, cycling, brisk walking, moderate walking, slow walking) was required. Moreover, participants were only included if they had ≥2 weekdays (Monday-Friday) and ≥1 weekend day (Saturday-Sunday) with valid accelerometer data.

Based on valid recording days, we calculated min/day for the different PA behaviors by dividing the sum of time in each PA behavior by the total number of valid recording days. The same was calculated for weekdays and weekend days separately. Time in PA was categorized into; 1) MVPA by adding the time spent in running, cycling, brisk walking and moderate walking per day, 2) light PA by adding the time spent in slow walking and standing per day, and 3) sedentary including time spent sitting per day. As we were focusing on behaviors during daytime, we did not include lying in the sedentary category as lying involves time spent in sleep. The volume of PA in each category was computed by multiplying min/day with the corresponding metabolic equivalent of task (MET) value for each PA behavior [24] (S1 Table).

#### Mediators

Trained examiners assessed gross motor abilities by using the Revised High-level Mobility Assessment Tool (HiMAT) [25, 26] and overall motor abilities by using the Bruininks Motor Ability Test (BMAT) Short Form [27]. The examiners were blinded to group, medical history, clinical characteristics, and results from previous follow-ups. An audit was carried out in both Finland and Norway before and during data collection to evaluate the protocol across the cohorts.

The Revised HiMAT is designed for adults and consists of eight items: walk, walk backward, walk on toes, walk over obstacle, run, skip, hop forward (more affected leg) and bound (less affected leg) [26]. Participants were instructed to perform the items as fast as possible over a distance of 10 meters, except for the bound item where participants were instructed to bound as long as possible, and the length was measured in cm. According to the manual we calculated standardized scores ranging from 0–4 for each item [26]. Failure, refusal or inability to perform the item was given a score of zero, and all item scores added up to a total score of 0–32 points.

The BMAT Short Form is designed for adults aged 40 years and older and consists of ten items combined into five subtests with the given point scores: Fine Motor Integration (0–14 points), Manual Dexterity (0–18 points), Coordination (0–10 points), Balance and Mobility (0–9 points) and Strength and Flexibility (0–18 points). Skipping or terminating an item was given a score of zero and each subtest score added up a total score of 0–69 points.

For both tests, a higher score indicates better motor abilities.

### Ethical considerations

The project was approved by the Ethics Committee IV of Helsinki University Hospital (HUS/1157) in Finland and the Regional Committee for Medical Research Ethics in Central-Norway (23879) and adhered to the Helsinki Declaration. Assessments were carried out between 11^th^ of September 2019 and 22^nd^ of January 2021. Written informed consent was obtained from all participants. Appointed doctors had medical responsibility during the data collection at each University Hospital. Participants received medically relevant feedback and were referred as appropriate if in need of further health services.

### Statistical analyses

IBM SPSS Statistics for Windows (version 28.0.0) was used for data analysis and a significance level of 0.05 was chosen. Mean differences of MET min/day and min of device-measured PA between the VLBW and control groups were estimated using linear regression with PA categories as dependent variable, group and cohort as fixed factor, and age and sex as covariates. We assessed normality of residuals by visual inspection of Q-Q plots. Due to some deviations from normality, bootstrapping with a bias-corrected and accelerated (BC_a_) method was applied with B = 2000 samples. Bootstrap confidence intervals are generally robust to deviations from the normal distribution assumption.

**Fig 2:**
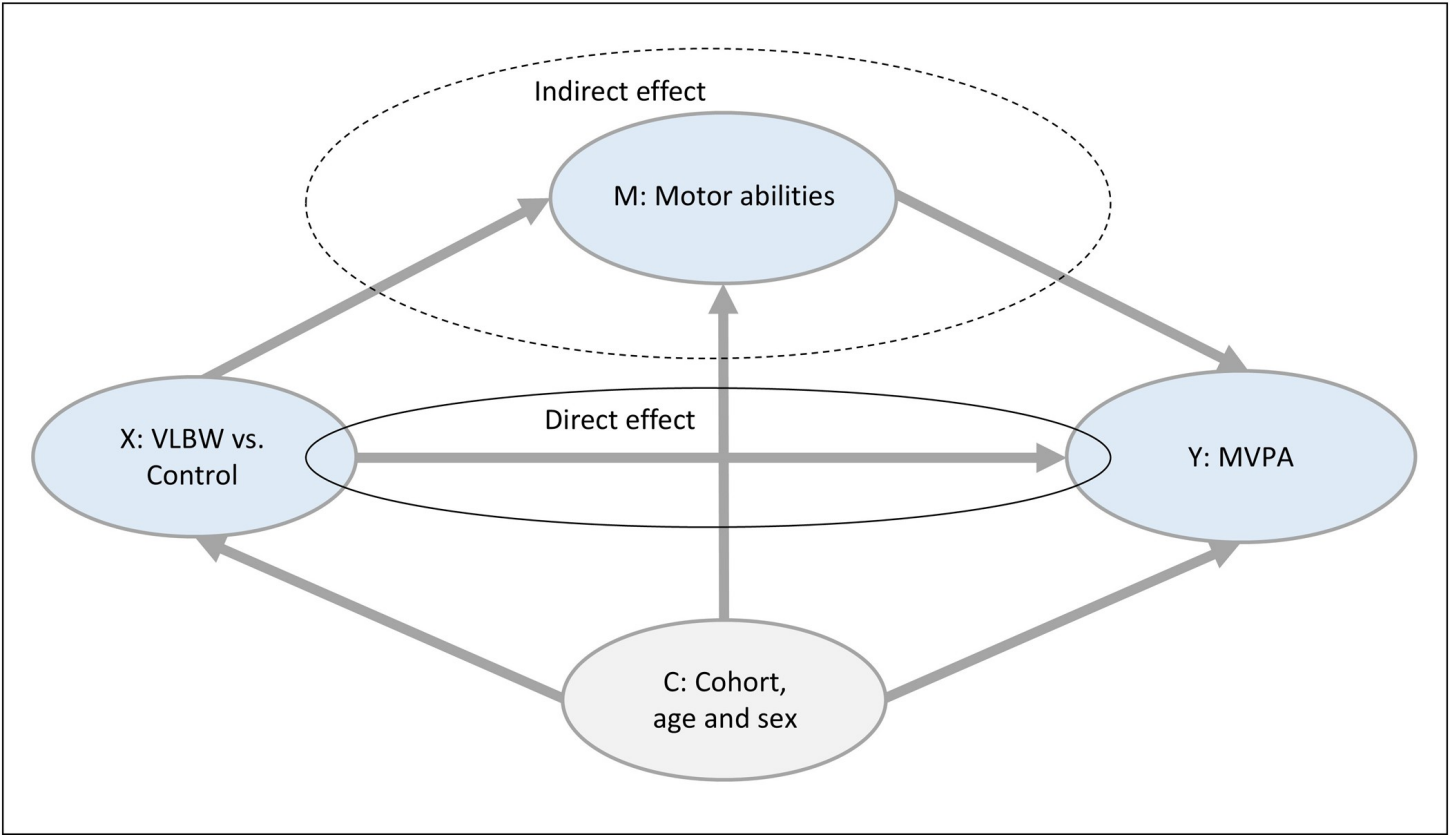
Relationship between group (X), motor abilities (M), and MVPA (Y) with confounders (C). Group: VLBW versus Control. Motor abilities: Total scores on the Revised High-level Mobility Assessment Tool or Bruininks Motor Ability Test Short Form. MVPA: Moderate to vigorous physical activity in metabolic equivalent of task in min/day. Abbreviation: VLBW = very low birth weight.

To explore whether VLBW was associated with MVPA, and the possible mediating role of motor abilities on this relationship, we performed mediation analyses by using PROCESS macro for IBM SPSS (www.processmacro.org) developed by Hayes with B = 5000 bootstrap samples. We entered VLBW as the predictor variable and MVPA in MET min/day as the outcome variable. The mediator variables Revised HiMAT total score and BMAT Short Form total score were added separately in two models (Fig 2). Bootstrap 95% CIs were generated for all indirect effects. Power calculations and non-participant analyses have been reported previously [9, 19].

### Supplementary analyses

We performed sensitivity analyses excluding participants with NSI. Separate analyses were performed for PA categories on weekdays and weekend days, as the time spent in PA has shown to differ between weekdays and weekend days in the general population [28].

Supplementary analyses with additional adjustment for maternal age, maternal and paternal education were performed as these were possible confounders. Additional adjustment for working hours/week was performed as it may affect time spent in PA. Due to possibly seasonal variations in PA, additional adjustment for the month of assessment defined by the first day of monitoring was performed [29]. All adjustment variables were entered as covariates in the regression analyses.

## Results

### Background and clinical characteristics

Table 1 shows background characteristics at birth and clinical characteristics in mid-adulthood of participants born with VLBW and controls.

**Table 1:** Background and clinical characteristics of the very low birth weight and control groups.

	VLBW			Control		
Background characteristics	n			n		
Maternal age at delivery (years), mean (SD)	87	30.0	(4.5)	108	30.8	(5.0)
Gestational age (weeks), mean (SD)	87	29.4	(2.3)	109	40.1	(1.1)
Birth weight (g), mean (SD)	87	1167	(207)	109	3685	(479)
Birth head circumference (cm), mean (SD)	83	26.6	(2.1)	94	35.3	(1.2)
Apgar score after 1 min, mean (SD)	74	6.3	(2.5)	93	8.9	(0.4)
Apgar score after 5 min, mean (SD)	57	7.8	(2.0)	46	9.7	(1.4)
Days of stay in NICU, mean (SD)	59	72.0	(47.7)	-	-	-
Days on ventilator, mean (SD)	84	7.6	(13.1)	-	-	-
Sepsis, n (%)	85	7	(8.2)	-	-	-
Interventricular hemorrhage, n (%)	68	10	(14.7)	-	-	-
Respiratory distress syndrome, n (%)	85	41	(48.2)	-	-	-
Bronchopulmonary dysplasia, n (%)	61	14	(23.0)	-	-	-
Maternal education, n (%)	84			102		
Basic or less		24	(28.6)		22	(21.6)
Secondary		16	(19.0)		22	(21.6)
Lower-level tertiary		27	(32.1)		27	(26.5)
Upper-level tertiary		17	(20.2)		31	(30.4)
Paternal education, n (%)	83			102		
Unknown		1	(1.2)		-	-
Basic or less		24	(28.9)		24	(23.5)
Secondary		19	(22.9)		25	(24.5)
Lower-level tertiary		21	(25.3)		20	(19.6)
Upper-level tertiary		18	(21.7)		33	(32.4)
**Clinical characteristics in mid-adulthood**	**n**			**n**		
Female, n (%)	87	49	(56.3)	109	60	(55.0)
Age, mean (SD)	87	36.3	(3.3)	109	35.8	(3.1)
Height (cm), mean (SD)	87	169.0	(9.5)	109	174.0	(9.6)
Weight (kg), mean (SD)	87	74.0	(17.2)	109	77.2	(15.0)
Body mass index (kg/m^2^), mean (SD)	87	25.9	(5.5)	109	25.4	(4.2)
CP, n (%)	87	3	(3.4)	109	1	(0.9)
NSI, n (%)	87	5	(5.7)	107	1	(0.9)
Education, n (%)	87			109		
Lower secondary or lower (ISCED 1–2)		4	(4.6)		2	(1.8)
Intermediate (ISCED 3–5)		39	(44.8)		31	(28.4)
Lower tertiary or higher (ISCED 6–8)		44	(50.6)		76	(69.7)
Working hours/week, mean (SD)	65	32.0	(12.5)	80	35.2	(11.7)
**Motor abilities in mid-adulthood**	**n**			**n**		
Revised High-level Mobility Assessment Tool total score, mean (SD)	85	23.5	(5.4)	109	27.2	(4.4)
Bruininks Motor Ability Test Short Form total score, mean (SD)	87	61.6	(8.2)	109	65.1	(3.2)

Abbreviations: CP = cerebral palsy; ISCED = International Standard Classification of Education; NICU = Neonatal intensive care unit; NSI = neurosensory impairment; SD = standard deviation; VLBW = very low birth weight.

### Device-measured physical activity

**Fig 3:**
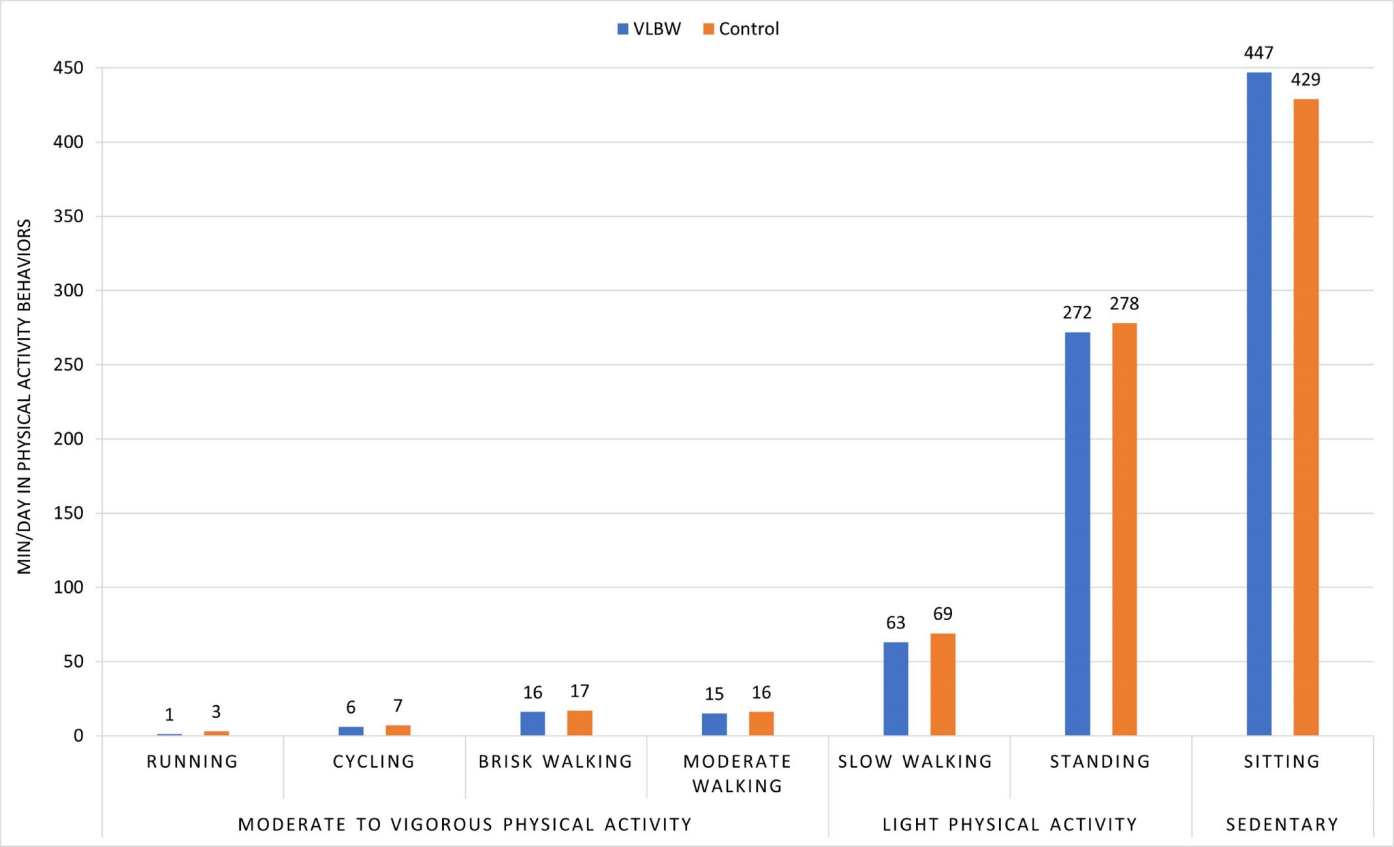
Min/day in physical activity behaviors in the very low birth weight and control groups. Abbreviation: VLBW = very low birth weight.

Fig 3 shows min/day of running, cycling, brisk walking, moderate walking, slow walking, standing and sitting in the two groups. The VLBW group spent 37.4 (SD 20.4) min/day in MVPA (Table 2), corresponding to approximately 262 min/week. In comparison, the control group spent 43.6 min/day (SD 24.1) in MVPA, corresponding to approximately 305 min/week.

**Table 2:** Min/day in physical activity categories in the very low birth weight and control groups.

	VLBW (n = 87)		Control (n = 109)		Mean difference (95% CI) adjusted for cohort, age and sex^a^	
	Mean	(SD)	Mean	(SD)		
MVPA	37.4	(20.4)	43.6	(24.1)	-7.3	(-13.9 to -1.0)
Light PA	335.4	(102.5)	347.2	(100.8)	-16.9	(-45.6 to 11.7)
Sedentary	446.7	(114.4)	429.2	(108.2)	20.3	(-9.1 to 49.1)

^a^Based on bias-corrected and accelerated bootstrap.

Abbreviations: CI = confidence interval; MVPA = moderate to vigorous physical activity; SD = standard deviation; VLBW = very low birth weight.

Table 3 shows MET min/day by PA categories: MVPA (running, cycling, brisk walking and moderate walking), light PA (slow walking and standing) and sedentary (sitting). MET min/day in MVPA was 149.3 (SD 83.7) in the VLBW group and 185.6 (SD 113.4) in the control group. The VLBW group spent 40.4 (95% CI: 13.3 to 69.4) MET min/day less in MVPA compared with the control group.

**Table 3:** Metabolic equivalent of task min/day in physical activity categories in the very low birth weight and control groups.

	VLBW (n = 87)		Control (n = 109)		Mean difference (95% CI) adjusted for cohort, age and sex^a^	
	Mean	(SD)	Mean	(SD)		
MVPA	149.3	(83.7)	185.6	(113.4)	-40.4	(-69.4 to -13.3)
Light PA	667.1	(206.8)	694.2	(198.8)	-37.3	(-94.1 to 18.4)
Sedentary	580.8	(148.7)	558.0	(140.7)	26.4	(-11.9 to 63.9)

^a^Based on bias-corrected and accelerated bootstrap.

Abbreviations: CI = confidence interval; MVPA = moderate to vigorous physical activity; SD = standard deviation; VLBW = very low birth weight.

### Moderate to vigorous physical activity and mediation by motor abilities

The unadjusted mean (SD) on the Revised HiMAT for the VLBW group was 23.5 (SD 5.4) points and 27.2 (SD 4.4) points for the control group. On the BMAT Short Form the unadjusted mean (SD) for the VLBW group was 61.6 (SD 8.2) points and 65.1 (SD 3.2) points for the control group (Table 1).

The mediation analyses were performed for MET min/day in MVPA only as MET min/day in light PA and sedentary did not differ between the groups. The results of the analyses are shown in Table 4. The total effect of gross motor abilities assessed by the Revised HiMAT was -40.2 (95% CI: -69.1 to -11.4) MET min/day. The direct effect was 24.6 (95% CI: -55.1 to 5.9) MET min/day. The indirect effect was -15.6 (95% CI: -28.5 to -5.4) MET min/day and statistically significant, indicating that part of the association between being born preterm with VLBW and less MVPA was mediated through poorer gross motor skills. The total effect of overall motor abilities assessed by the BMAT Short Form on MET min/day in MVPA was -40.4 (95% CI: -69.0 to -11.9). The direct effect was -38.1 (95% CI: -67.8 to -8.5) MET min/day, while the indirect effect was very small (-2.3, 95% CI: -8.2 to 3.6) and not statistically significant.

**Table 4:** Direct, indirect and total effect of the very low birth weight group on metabolic equivalent of task min/day of moderate to vigorous physical activity with motor abilities as a mediator.

Motor abilities	n^a^	Direct effect of VLBW		Indirect effect of VLBW		Total effect of VLBW	
		Estimate	(95% CI)	Estimate	(95% CI)	Estimate	(95% CI)
Revised HiMATtotal score	85, 109	-24.6	(-55.1 to 5.9)	-15.6	(-28.5 to -5.4)	-40.2	(-69.1 to -11.4)
BMAT Short Form total score	87, 109	-38.1	(-67.8 to -8.5)	-2.3	(-8.2 to 3.6)	-40.4	(-69.0 to -11.9)

^a^VLBW, Control.

Analyses adjusted for cohort, age and sex.

Abbreviations: BMAT = Bruininks Motor Ability Test; CI = confidence interval; HiMAT = High-level Mobility Assessment Tool; VLBW = very low birth weight.

### Supplementary analyses

The differences in MET min/day in PA were essentially the same in sensitivity analyses when participants with neurosensory impairment (NSI) were excluded (S2 Table). When analyzed separately for weekdays and weekend days (S3 Table), the VLBW group spent 64.7 (95% CI: 17.6 to 111.8) MET min/day more in sedentary and 85.7 (95% CI: 23.0 to 150.2) MET min/day less in light PA during the weekend compared with the control group.

Results barely changed when additionally adjusted for month of assessment (S4 Table) or for maternal age, maternal and paternal education (S5 Table). Likewise, additional adjustment for working hours/week among participants who were working during the monitoring period had minor influence on the group difference in MET min/day of MVPA (S6 Table) even though the upper limit of the CI was just above zero.

## Discussion

### Main findings

In this unique joint assessment of two birth cohorts from Finland and Norway, we found that adults born with VLBW had less device-measured MET min/day in MVPA than controls born at term. Poorer gross motor abilities measured by the Revised HiMAT partly explained the association between being born with VLBW and having less MET min/day in MVPA.

### Strengths and limitations

The participants born with VLBW have been longitudinally followed up with comprehensive assessments of physical and mental health into mid-adulthood. Even though the participants from the HeSVA cohort were born slightly earlier (1978–1985) than the participants from the NTNU cohort (1986–1988), we consider the temporality effects to be minor as all participants were born before the surfactant era of the 1990s. In addition, we adjusted for cohort in our analyses.

Loss to follow-up is common in cohort studies [30]. In our study, the participation rate based on the invited was 55% in the VLBW group and 59% in the control group. A potential reason for this loss to follow-up could be that the data collection took place partly during the COVID-19 pandemic, hindering some of the participants from meeting in person.

A strength of the study was the use of two accelerometers in a comprehensive assessment of PA behaviors in free-living settings with recording days of >23 hours. We utilized the XGBoost machine learning model, which has a high accuracy in detecting free-living PA behaviors based on the dual setup of accelerometers on the thigh and back [22, 23]. The classification of PA was restricted to the seven specific behaviors of running, cycling, brisk walking, moderate walking, slow walking, standing and sitting. As the algorithms are continuously developing, the model has potential to become even more accurate and cover more PA behaviors in the future. It is reasonable to assume that the potential bias of participants increasing their PA beyond their usual level as response to being monitored would have been present in both groups. However, as this is most likely to occur in the beginning of the monitoring period, it was partly accounted for as the first day of recording was removed for all participants.

Another strength of the study was that motor abilities were assessed by trained examiners blinded to group, medical history, clinical characteristics and results from previous follow-ups. Furthermore, we used standardized motor tests. However, there is a lack of validated assessment tools measuring overall motor abilities in young to mid-adulthood. Whereas the HiMAT Revised may be used for adults of all ages [26], the BMAT Short Form is designed for adults aged 40 years and older [27]. As a result, the test might have been too easy to perform for participants in our study. This could have affected the test’s ability to differentiate motor skills, potentially underestimating group differences and the mediating effect of motor abilities on MVPA.

### Consistency with literature

Our finding of group differences in device-measured MET min/day in MVPA between adults born VLBW and controls are novel. Previously, Tikanmäki et al. [31] and Kaseva et al. [32] did not find lower PA among young adults born early preterm (<34 weeks) in the Finnish ESTER cohort at 23 years or among adults born with VLBW in the HeSVA cohort at 25 years. While we assessed PA by two tri-axial accelerometers on the back and thigh and required >23 valid hours of recording per day, PA was assessed by a single hip-worn or a wrist-worn accelerometer for a minimum of 8.3 and 10 hours per day respectively, in the two studies. This may have given less accurate measurements and could explain the findings of no group difference.

Studies using self-reported PA have shown that adults born with VLBW have less time in PA compared with controls. In young adulthood, participants born with VLBW in the HeSVA cohort reported less leisure time conditioning PA [11, 12], including less time in higher intensities for a shorter duration of time compared with controls born at term [33]. This is also consistent with another study from the ESTER cohort, where adults born early preterm reported a considerably lower volume of PA in MET hours/year compared with controls [13]. Furthermore, Yang et al. [10] found a difference in reported number of days in MVPA over the past week among adults born with VLBW compared with controls, even though they did not report time spent per day, which may affect the accuracy of the PA measurement. Also, another recent individual participant data meta-analysis of five cohorts, including HeSVA and NTNU LBW Life, found approximately one hour/week less time in self-reported MVPA in the very preterm/VLBW group compared with the control group [14].

In the joint HeSVA-NTNU study, we have previously reported that adults born with VLBW have poorer overall, fine and gross motor abilities compared with controls [19]. To our knowledge, this is the first study to show that gross motor abilities mediate the relationship between VLBW and device-measured MET min in MVPA in adulthood. However, a study of exercise capacity in children aged 11–13 years with extremely low birth weight (ELBW) found that motor coordination measured by the Movement Assessment Battery for Children was the strongest predictor of exercise capacity both among the children born ELBW and controls [34].

We found that gross motor abilities, measured by the Revised HiMAT, mediated the association between being born with VLBW and MVPA. Even if the direct effect of VLBW on MVPA was larger than the indirect effect for the model with Revised HiMAT, only the latter was significant. As the items of the Revised HiMAT are mainly based on locomotion, they may correspond to PA in everyday life. In contrast, overall motor abilities as measured by the BMAT Short Form, was not a mediator. The BMAT Short Form assesses a broader specter of motor abilities, including fine motor integration and manual dexterity, which may be less important for PA.

A large study of children and adults in the general US population reported more overall PA during weekdays than during weekend days [28]. When we performed separate analyses for weekdays and weekend days, the group difference in MVPA was significant during weekdays, and barely not significant during weekend days. However, the VLBW group had less MET min/day in light PA and more MET min/day in sedentary during weekend days. In sum, our findings indicate less PA in the VLBW group regardless of weekdays or weekend days.

### Biological plausibility

Being born preterm with VLBW has implications for the development of organ systems, particularly affecting the brain and respiratory system [3]. These effects can have long-term consequences, including neurodevelopmental disorders and impaired cardiorespiratory function. In this study, we used adults born preterm with VLBW as a model of early life origins of PA. We focused on motor abilities as a possible explanation of less MVPA in adults born with VLBW. Indeed, we found that poorer gross motor abilities partly explained the association between being born with VLBW and engaging less in MVPA.

Some studies have suggested that poorer lung function may be associated with less PA [13, 35]. In a recent nationwide register study from Finland and Norway, being born preterm was associated with increased risk of asthma and chronic obstructive pulmonary disease during adulthood, and this association was stronger among individuals with BPD [36]. However, previous findings from the HeSVA cohort [11, 12] indicate that self-reported PA do not differ according to BPD or asthma status. Thus, the lower PA in individuals born preterm is probably not explained by their lower lung function.

### Clinical implications

In our study, we focused on PA in the moderate and high intensities which gives the most health benefits [1]. Even if both groups met the recommendations by The World Health Organization from 2020 of accumulating at least 150–300 min of moderate PA, 75–150 min of vigorous PA, or an equivalent combination of both moderate PA and vigorous PA per week [1], we still found that the VLBW group engaged less in MVPA. Increasing PA by even small amounts, especially in higher intensities, is beneficial and clinically relevant [37] and the VLBW group might have an additional health benefit of being physically active in MVPA as they are at higher risk of adverse health outcomes due to being born preterm with VLBW. Interventions to increase PA and particularly MVPA, with focus on challenging gross motor skills, could improve the health and long-term outcomes of individuals born with VLBW [16]. A previous study by Saigal et al. [38] found that young adults born with ELBW had lower physical self-efficacy, perceived physical ability and physical self-competence compared with controls. It is reasonable to believe that engaging in exercise programs for individuals born preterm may potentially increase perceived physical ability and thus make PA even more rewarding for this population.

Early interventions may be especially beneficial, as the development of motor skills is most prominent during childhood. One example of such an intervention is the Dance PREEMIE, a dance participation intervention for children born extremely preterm/ELBW with motor impairment at preschool age, in which dance teachers were provided with physiotherapy training to promote PA participation [39]. It was proven feasible [40] and acceptable [41] to both the parents of participating children and the dance teachers in a small sample, yet its efficacy in larger samples is still to be determined. Similar interventions that provide children the opportunity to develop and perform motor skills in a real-world environment together with peers may be advantageous.

## Conclusion

In conclusion, we found that adults born with VLBW had less MVPA than controls measured by accelerometry. Gross motor abilities mediated the association between being born with VLBW and MVPA. Combining device-measured PA and standardized motor tests has given new knowledge of the relationship between physical and motor function among adults born with VLBW, which may be used in initiatives and programs aiming at promoting exercise and enhancing physical self-confidence in this group.

## Supporting information

S1 Table Metabolic equivalent of task values of physical activity behaviors. Abbreviations: MET = metabolic equivalent of task.
(DOCX)

S2 Table Metabolic equivalent of task min/day in physical activity categories in the very low birth weight and control groups when participants with neurosensory impairment were excluded. ^a^Based on bias-corrected and accelerated bootstrap. Abbreviations: CI = confidence interval; MVPA = moderate to vigorous physical activity; SD = standard deviation; VLBW = very low birth weight.
(DOCX)

S3 Table Metabolic equivalent of task min/day in physical activity categories in the very low birth weight and control groups for weekdays and weekend days separately. ^a^Based on bias-corrected and accelerated bootstrap. Abbreviations: CI = confidence interval; MVPA = moderate to vigorous physical activity; SD = standard deviation; VLBW = very low birth weight.
(DOCX)

S4 Table Metabolic equivalent of task min/day in physical activity categories in the very low birth weight and control groups with additional adjustment for month of assessment. ^a^Based on bias-corrected and accelerated bootstrap. Abbreviations: CI = confidence interval; MVPA = moderate to vigorous physical activity; SD = standard deviation; VLBW = very low birth weight.
(DOCX)

S5 Table Metabolic equivalent of task min/day in physical activity categories in the very low birth weight and control groups with additional adjustment for maternal age, maternal and paternal education. ^a^Based on bias-corrected and accelerated bootstrap. Abbreviations: CI = confidence interval; MVPA = moderate to vigorous physical activity; SD = standard deviation; VLBW = very low birth weight.
(DOCX)

S6 Table Metabolic equivalent of task min/day in physical activity categories in the very low birth weight and control groups with additional adjustment for hours/week among participants who were working during the monitoring period. ^a^Based on bias-corrected and accelerated bootstrap. Abbreviations: CI = confidence interval; MVPA = moderate to vigorous physical activity; SD = standard deviation; VLBW = very low birth weight.
(DOCX)
