# Supplementary material for: Device-measured physical activity in adults born preterm with very low birth weight and mediation by motor abilities
Source: PLoS One. 2025 Jan 7;20(1):e0312875. doi: 10.1371/journal.pone.0312875 (PMC11706474; doi:10.1371/journal.pone.0312875)
Supplement: S1 Table — Abbreviations: MET = metabolic equivalent of task. (DOCX) [file pone.0312875.s001.docx]

**S1 Table.** **Metabolic equivalent of task values of physical activity behaviors.**

| **2011 Compendium (1)** | | **Activity** | **Description** |
| --- | --- | --- | --- |
| Codes | METs |  |  |
| 12030 | 8.3 | Running | *Running, 5 mph (12 min/mile)* |
| 01010 | 4.0 | Cycling | *Bicycling, <10 mph, leisure, to work or for pleasure (Taylor Code 115)* |
| 17200 | 4.3 | Brisk walking | *Walking, 3.5 mph, level, brisk, firm surface, walking for exercise* |
| 17190 | 3.5 | Moderate walking | *Walking, 2.8 to 3.2 mph, level, moderate pace, firm surface* |
| 17152 | 2.8 | Slow walking | *Walking, 2.0 mph, level, slow pace, firm surface* |
| 07041 | 1.8 | Standing | *Standing, fidgeting* |
| 07021 | 1.3 | Sitting | *Sitting quietly, general* |

Abbreviations: MET=metabolic equivalent of task.

Reference

1. B. E. Ainsworth *et al.*, 2011 Compendium of Physical Activities: a second update of codes and MET values. *Med Sci Sports Exerc* **43**, 1575-1581 (2011).
