# Supplementary material for: Device-measured physical activity in adults born preterm with very low birth weight and mediation by motor abilities
Source: PLoS One. 2025 Jan 7;20(1):e0312875. doi: 10.1371/journal.pone.0312875 (PMC11706474; doi:10.1371/journal.pone.0312875)
Supplement: S2 Table — aBased on bias-corrected and accelerated bootstrap. Abbreviations: CI = confidence interval; MVPA = moderate to vigorous physical activity; SD = standard deviation; VLBW = very low birth weight. (DOCX) [file pone.0312875.s002.docx]

**S2 Table.** **Metabolic equivalent of task min/day in physical activity categories in the very low birth weight and control groups when participants with neurosensory impairment were excluded.**

|  | **VLBW (n=82)** | | **Control (n=108)** | | **Mean difference (95% CI)**  **adjusted for cohort, age and sex^a^** | |
| --- | --- | --- | --- | --- | --- | --- |
|  | **Mean** | **(SD)** | **Mean** | **(SD)** |  |  |
| MVPA | 150.0 | (85.3) | 185.4 | (113.9) | -39.6 | (-69.1 to -9.7) |
| Light PA | 669.8 | (211.0) | 694.6 | (199.6) | -36.0 | (-90.8 to 20.1) |
| Sedentary | 573.7 | (147.5) | 557.3 | (141.2) | 20.9 | (-15.9 to 58.3) |

^a^Based on bias-corrected and accelerated bootstrap.

Abbreviations: CI=confidence interval; MVPA=moderate to vigorous physical activity; SD=standard deviation; VLBW=very low birth weight.
