# Supplementary material for: Device-measured physical activity in adults born preterm with very low birth weight and mediation by motor abilities
Source: PLoS One. 2025 Jan 7;20(1):e0312875. doi: 10.1371/journal.pone.0312875 (PMC11706474; doi:10.1371/journal.pone.0312875)
Supplement: S3 Table — aBased on bias-corrected and accelerated bootstrap. Abbreviations: CI = confidence interval; MVPA = moderate to vigorous physical activity; SD = standard deviation; VLBW = very low birth weight. (DOCX) [file pone.0312875.s003.docx]

**S3 Table. Metabolic equivalent of task min/day in physical activity categories in the very low birth weight and control groups for weekdays and weekend days separately.**

|  | **VLBW (n=87)** | | **Control (n=109)** | | **Mean difference (95% CI)**  **adjusted for cohort, age and sex^a^** | |
| --- | --- | --- | --- | --- | --- | --- |
|  | **Mean** | **(SD)** | **Mean** | **(SD)** |  |  |
| **Weekdays** |  |  |  |  |  |  |
| MVPA | 157.9 | (93.0) | 197.5 | (120.3) | -43.6 | (-75.6 to -13.8) |
| Light PA | 699.4 | (240.6) | 707.1 | (226.3) | -17.4 | (-82.3 to 52.9) |
| Sedentary | 581.0 | (162.9) | 578.2 | (169.7) | 5.4 | (-39.4 to 49.9) |
| **Weekend days** |  |  |  |  |  |  |
| MVPA | 130.3 | (108.3) | 162.9 | (147.1) | -35.7 | (-71.2 to -0.6) |
| Light PA | 600.5 | (229.0) | 676.5 | (219.2) | -85.7 | (-150.2 to -23.0) |
| Sedentary | 575.6 | (186.9) | 517.3 | (149.8) | 64.7 | (17.6 to 111.8) |

^a^Based on bias-corrected and accelerated bootstrap.

 Abbreviations: CI=confidence interval; MVPA=moderate to vigorous physical activity; SD=standard deviation; VLBW=very low birth weight.
