# Supplementary material for: Device-measured physical activity in adults born preterm with very low birth weight and mediation by motor abilities
Source: PLoS One. 2025 Jan 7;20(1):e0312875. doi: 10.1371/journal.pone.0312875 (PMC11706474; doi:10.1371/journal.pone.0312875)
Supplement: S4 Table — aBased on bias-corrected and accelerated bootstrap. Abbreviations: CI = confidence interval; MVPA = moderate to vigorous physical activity; SD = standard deviation; VLBW = very low birth weight. (DOCX) [file pone.0312875.s004.docx]

**S4 Table. Metabolic equivalent of task min/day in physical activity categories in the very low birth weight and control groups with additional adjustment for month of assessment.**

|  | **VLBW (n=87)** | | **Control (n=109)** | | **Mean difference (95% CI) adjusted for cohort, age and sex^a^** | | **Mean difference (95% CI) adjusted for cohort, age, sex and month** | |
| --- | --- | --- | --- | --- | --- | --- | --- | --- |
|  | **Mean** | **(SD)** | **Mean** | **(SD)** |  |  |  |  |
| MVPA | 149.3 | (83.7) | 185.6 | (113.4) | -40.4 | (-69.4 to -13.3) | -41.7 | (-70.3 to -13.0) |
| Light PA | 667.1 | (206.8) | 694.2 | (198.8) | -37.3 | (-94.1 to 18.4) | -34.6 | (-90.0 to 21.1) |
| Sedentary | 580.8 | (148.7) | 558.0 | (140.7) | 26.4 | (-11.9. to 63.9) | 26.4 | (-11.4 to 64.5) |

^a^Based on bias-corrected and accelerated bootstrap.

Abbreviations: CI=confidence interval; MVPA=moderate to vigorous physical activity; SD=standard deviation; VLBW=very low birth weight.
