# Supplementary material for: Device-measured physical activity in adults born preterm with very low birth weight and mediation by motor abilities
Source: PLoS One. 2025 Jan 7;20(1):e0312875. doi: 10.1371/journal.pone.0312875 (PMC11706474; doi:10.1371/journal.pone.0312875)
Supplement: S5 Table — aBased on bias-corrected and accelerated bootstrap. Abbreviations: CI = confidence interval; MVPA = moderate to vigorous physical activity; SD = standard deviation; VLBW = very low birth weight. (DOCX) [file pone.0312875.s005.docx]

**S5 Table. Metabolic equivalent of task min/day in physical activity categories in the very low birth weight and control groups with additional adjustment for maternal age, maternal and paternal education.**

|  | **VLBW (n=82)** | | **Control (n=100)** | | **Mean difference (95% CI) adjusted for cohort, age and sex^a^** | | **Mean difference (95% CI) adjusted for cohort, age, sex, maternal age, maternal and paternal education^a^** | |
| --- | --- | --- | --- | --- | --- | --- | --- | --- |
|  | **Mean** | **(SD)** | **Mean** | **(SD)** |  |  |  |  |
| MVPA | 149.0 | (79.5) | 186.7 | (115.6) | -40.5 | (-69.3 to -11.4) | -35.5 | (-64.5 to -6.3) |
| Light PA | 676.4 | (202.1) | 695.5 | (184.0) | -25.6 | (-76.3 to 25.2) | -24.4 | (-72.7 to 28.1) |
| Sedentary | 588.9 | (146.6) | 555.4 | (140.2) | 36.0 | (-2.5 to 75.1) | 36.2 | (-3.8 to 77.4) |

^a^Based on bias-corrected and accelerated bootstrap.

Abbreviations: CI=confidence interval; MVPA=moderate to vigorous physical activity; SD=standard deviation; VLBW=very low birth weight.
