# Supplementary material for: Device-measured physical activity in adults born preterm with very low birth weight and mediation by motor abilities
Source: PLoS One. 2025 Jan 7;20(1):e0312875. doi: 10.1371/journal.pone.0312875 (PMC11706474; doi:10.1371/journal.pone.0312875)
Supplement: S6 Table — aBased on bias-corrected and accelerated bootstrap. Abbreviations: CI = confidence interval; MVPA = moderate to vigorous physical activity; SD = standard deviation; VLBW = very low birth weight. (DOCX) [file pone.0312875.s006.docx]

**S6 Table. Metabolic equivalent of task min/day in physical activity categories in the very low birth weight and control groups with additional adjustment for hours/week among participants who were working during the monitoring period.**

|  | **VLBW (n=65)** | | **Control (n=80)** | | **Mean difference (95% CI)**  **adjusted for cohort, age and sex^a^** | | **Mean difference (95% CI) adjusted for cohort, age, sex and working hours^a^** | |
| --- | --- | --- | --- | --- | --- | --- | --- | --- |
|  | **Mean** | **(SD)** | **Mean** | **(SD)** |  |  |  |  |
| MVPA | 159.1 | (80.9) | 189.5 | (109.3) | -34.0 | (-65.6 to -1.4) | -31.9 | (-63.2 to 0.1) |
| Light PA | 689.7 | (196.2) | 702.0 | (183.6) | -22.9 | (-80.0 to 33.6) | -16.3 | (-71.8 to 40.9) |
| Sedentary | 583.0 | (144.3) | 575.7 | (135.0) | 13.6 | (-26.1 to 51.6) | 16.4 | (-22.5 to 55.0) |

^a^Based on bias-corrected and accelerated bootstrap.

Abbreviations: CI=confidence interval; MVPA=moderate to vigorous physical activity; SD=standard deviation; VLBW=very low birth weight.
